# Is punctate palmoplantar keratoderma type 1 associated with malignancy? A systematic review of the literature

**DOI:** 10.1186/s13023-023-02862-8

**Published:** 2023-09-13

**Authors:** S. B. Gram, J. Bjerrelund, A. M. Jelsig, A. Bygum, C. Leboeuf-Yde, L. B. Ousager

**Affiliations:** 1 Department of Clinical Genetics, Odense University Hospital, J.B. Winsløws Vej 4, Indgang 24, 5000 Odense C, Denmark; 2 Department of Clinical Research, University of Southern Denmark, Odense, Denmark; 3 Department of Clinical Genetics, Copenhagen University Hospital, Copenhagen, Denmark; 4 Institute for Regional Health Research, University of Southern Denmark, Odense, Denmark; 5 Hudklinikken Kolding, Kolding, Denmark

**Keywords:** Punctate palmoplantar keratoderma type 1, Malignancy, *AAGAB*

## Abstract

**Background:**

An association between punctate palmoplantar keratoderma type 1 (PPPK1) and malignancy has been proposed for decades. Some authors suggest that individuals with PPPK1 should undergo screening for various types of malignancies while others caution that an association is not well-established. In this systematic review, we summarized and evaluated the current evidence for a possible association between PPPK1 and malignancy.

**Methods:**

The review was conducted along PRISMA guidelines. The search used Embase, MEDLINE, Scopus, and the Human Gene Mutation Database up to March 2022. All studies reporting on individuals with the diagnosis of PPPK1 with *or* without history of malignancy were included. Two authors screened for eligible studies, extracted predefined data, and performed a quality assessment.

**Results:**

Of 773 studies identified, 45 were included. Most studies were reports on single families (24 of 45 studies) or multiple families (10 of 45 studies). The number of index cases with PPPK1 across all included studies was 280, and when family members reported with PPPK1 were added, a total of 817 individuals were identified. Overall, 23 studies reported on individuals with PPPK1 with a history of malignancy, whereas 22 studies reported on individuals with PPPK1 without a history of malignancy. Although the extracted data were not considered to be of sufficient quality to synthesize and answer our research question, the review did not confirm an association between PPPK1 and malignancy.

**Conclusion:**

This review shows that there is a lack of well-designed studies on this topic to conclude whether individuals with PPPK1 have an increased risk of malignancy. Based on the present literature, however, we could not confirm an association between PPPK1 and malignancy and find it highly questionable if patients with PPPK1 should be offered surveillance for malignancies.

**Supplementary Information:**

The online version contains supplementary material available at 10.1186/s13023-023-02862-8.

## Introduction

Palmoplantar keratoderma (PPK) is a heterogeneous group of rare disorders characterized by hyperkeratinization of the skin on the palms and soles. The disease can either be hereditary due to pathogenic variants in disease-causing genes or acquired due to e.g., arsenic exposure, menopause, and paraneoplastic syndromes. PPK is most often an isolated skin disease, but can also be associated with extracutaneous manifestations such as hearing loss, growth delay, and heart disease [1, 2]. PPK is categorised into different subtypes based on the pattern of hyperkeratosis, including punctate, diffuse, focal, and striate PPK. Isolated punctate PPK can be further subdivided into three variants: Buschke–Fischer–Brauer disease (type 1), porokeratosis punctata palmaris et plantaris (type 2), and acrokeratoelastoidosis (type 3). Punctate PPK may also be part of the clinical picture in several syndromes with extracutaneous symptoms, such as Cowden syndrome, Cole disease, and Darier disease.

The genetic background for punctate PPK type 1 (PPPK1) was identified in 2012, when a disease-causing variant in *AAGAB* [3] was reported. More than 49 disease-causing variants have since been identified in *AAGAB*. Although a disease-causing variant in *COL14A1* was identified in a single Chinese family with PPPK1 [4], it has not been possible to detect pathogenic variants in *COL14A1* in other families with this clinical phenotype. Likewise, not all patients with a PPPK1 phenotype have a variant in *AAGAB* identified. This has led to the hypothesis of additional, as yet unidentified, causative genes [5, 6]. Still, pathogenic variants in *AAGAB* are considered the major cause of the PPPK1.

Other subtypes of PPK have been described as being associated with increased risk of malignancy, e.g. a subtype of focal PPK named *Tylosis with oesophageal cancer* (OMIM: 148500), where patients have a very high lifetime risk of oesophageal cancer [7]. A similar association between punctate PPK type 1 and various types of malignancies, especially gastrointestinal cancers, has long been suggested [8, 9]. This has led several authors to propose surveillance for malignancy in all individuals with PPPK1 [8, 10–14]. The suggested association between PPPK1 and malignancy is not well-established, making it difficult for clinicians to manage and counsel these patients.

The aim of this systematic review was to summarize the current evidence of a possible association between punctate palmoplantar keratoderma type 1 and malignancy.

## Methods

The systematic review was performed according to the preferred reporting items for systematic reviews and meta-analyses (PRISMA) guidelines [15]. The review followed a protocol that was discussed and prepared by all authors and contained items considered relevant for the research question. Two authors (SG, JB) independently screened for eligible studies, extracted data from the included studies, and performed quality assessment. Their findings were compared, and any disagreements were discussed. If necessary, discrepancies were solved in dialogue with a third author (LBO). Data extraction and quality assessment used a predesigned form based on the protocol. The protocol and later minor changes are available from the authors on request.

### Search strategy and information sources

The search was performed on 7 October 2020 and updated on 24 January 2022 using the search engines Embase, MEDLINE, and Scopus. Search terms were ((palm* OR plantar* OR palmoplantar*) AND (kerato* OR hyperkerato*) AND punct*) OR (Buschke and Fischer and Brauer) OR *AAGAB* OR *COL14A1*. Terms were searched as free-text terms and as subject headings when available (Emtree and MeSH terms). In addition, we searched for disease-causing variants in *AAGAB* and *COL14A1* in the Human Gene Mutation Database (HGMD) [16]. Languages were restricted to English, Danish, Swedish, Norwegian and German. No restriction was placed on date of publication. Grey literature databases were not searched, but some of the selected databases included, for example, conference abstracts. After screening was completed, the reference lists of the included studies were screened. This led to identification of two additional search terms (‘papulos*’ and ‘dissemina*’). Therefore, a supplementary search was performed on 1 March 2022 with the search terms ((palm* OR plantar* OR palmoplantar*) AND (kerato* OR hyperkerato*) AND papulos*) and ((palm* OR plantar* OR palmoplantar*) AND (kerato* OR hyperkerato*) AND dissemina*). The search strategy was established and performed in collaboration with a health science research librarian.

### Eligibility criteria and screening process

Studies were considered eligible if they met the following inclusion criterion: Any type of study design reporting on individuals with the diagnosis of PPPK1 with *or* without a history of malignancy. Exclusion criterion was any signs of PPPK1 being an acquired skin disease. The screening process was handled using the platform Covidence [17]. First, the selection criteria were applied to the title and abstract. Second, decisions about eligibility for inclusion were made after full-text screening.

### Data items and collection process

The following characteristics of the included studies were obtained: name of first author, year of publication, and study design. Study design was categorized as (i) single cases (no information about family history or no family members with PPPK1), (ii) family report (at least two family members with PPPK1), (iii) family reports (reports on several families with PPPK1), or (iv) cohort studies (reports on multiple individuals with PPK but no specific information about affected family members). We extracted the following information about *index cases* with PPPK1 *(the patient that initially drew medical attention in the family)*: number of index cases, age, sex, ethnicity and/or nationality, and clinical or molecular genetic diagnosis. Information on consanguinity and disease duration of PPPK1 for index patients were noted. Finally, we gathered the results of each study relating to our research question: number of individuals with PPPK1, and number of individuals with PPPK1 and malignancies. For studies reporting on individuals with PPPK1 with malignancies: cancer type, sex and age at diagnosis were extracted.

### Assessment of methodological quality

As no standardized, validated quality assessment tool was considered suitable for the topic or the most frequent study types (family report/reports), we designed a quality assessment tool for our research questions (Additional file 1: Table S1). Our pre-hoc minimal criteria were the following: representativeness of the study population (criterion A), quality of information about family members (criterion B, divided into criteria B1 and B2), and presence of a comparison group in relation to the risk of malignancy (criterion C). We evaluated these criteria using 8 sub-criteria (Additional file 1: Table S1) with assessment options of ‘yes’, ‘perhaps’, and ‘no’, with the addition of ‘not relevant’ for criteria B1 and B2. For ease of interpretation, we colour-coded the results using green for ‘yes’, orange for ‘perhaps’, red for ‘no’, and grey for ‘not relevant’. Based on the quality assessment, we rated the general impression of each study in regard to how useful it was for answering our research question, i.e. good (green), fair (yellow), or poor (red). Our intention was to rely only on studies judged to be of acceptable quality based on our assessment tool.

## Results

### Study selection

**Fig. 1:**
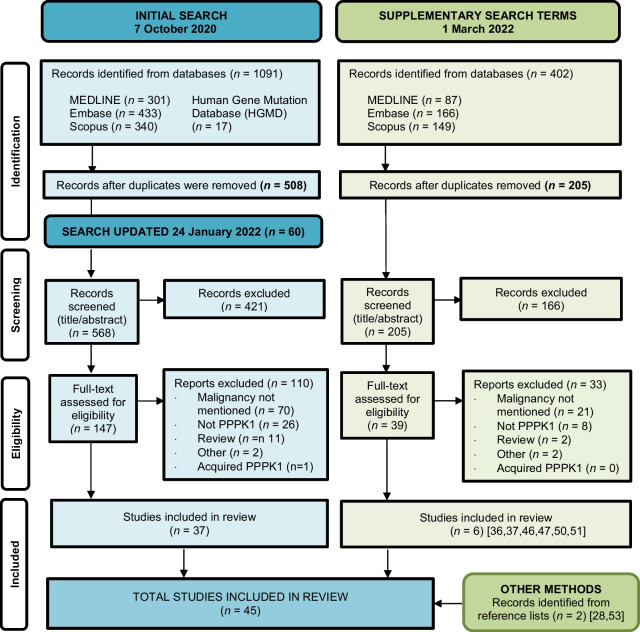
PRISMA flow diagram of search results and selection process of articles

The search generated 773 records, including the results of the initial search (*n* = 508), the updated search (*n* = 60), and the supplementary search with two extra search terms (*n* = 205). Manual search of the reference lists yielded two more studies. After the screening process, 45 studies were included for analysis. An overview of the results of the literature search, the study selection process, and reasons for exclusions is shown in Fig. 1.

### Study characteristics and description of extracted data

The characteristics of the included studies and extracted data are listed in Table 1 and Additional file 1: Tables S2/S3. The study types and approaches were quite heterogeneous, consisting of family report/reports (*n* = 34), single cases (*n* = 6), cohort studies (*n* = 3), and a mixture of single cases and family reports (*n* = 2). The study populations (based on the information about index cases) varied regarding sex, age,, and nationality/ethnicity, indicating that the literature was based on a potentially representative population of individuals with PPPK1.

**Table 1:** Study characteristics and description of extracted data in the 45 included studies

Author	Year	Study design	Description of index cases					Number of individuals in the study reported with	
		(no. of families)	Number of index cases	Sex (M/F) (no. of cases)	Age (age in years)	Ethnicity/ nationality	Clinical (C) or molecular genetic (M) diagnosis (no. of cases) (*gene*)	PPPK1	PPPK1 and malignancy
Elhaji [6]	2020	Family reports (18)	18	M(8), F(10)	NA	Canadian	C(7), M(11) (*AAGAB*)	52	9
Johnston [18]^a^	2020	Family report	1	F	80	NA	M (*AAGAB*)	> 1	1
Charfeddine [12]	2016	Family reports (8)	18	M(11), F(7)	21–95	Tunisian	M (*AAGAB*)	62	1
Podder [19]	2015	Family report	1	M	66	NA	C	6	1
Elleuch [20]	2014	Family report	1	M	70	NA	C	38	3
Cui [5]	2013	Family reports (8) Single cases	8 28	M(6), F(2) NA	NA	Chinese	C(3),M(5)* (AAGAB)* C(27),M(1) (*AAGAB*)	57 28	5 3
Pöhler [21]	2013	Family reports (6)	5^b^	M(1), F(4)	42–79	Scottish(4), English(1), Mexican(1)	M(*AAGAB*)	44	1
Kiritsi [22]	2013	Family reports (3)	4	F(2), NA(2)	46–75	NA	M (*AAGAB*)	> 8	1
Vinod [23]	2012	Family report	1	M	65	NA	C	7	1
Pohler [24]	2012	Family reports (18)	17^b^	M(5), F(12)	NA	Scottish(12), Irish(1), Japanese(2), Tunisian(3)	M (*AAGAB*)	93	“a few”
O'Toole [25]	2012	Family report	1	F	54	NA	C	2	1
Mamai [26]	2012	Family reports (3)	1^b^	M	NA	Tunisian	C	54	?^c^
Guo [4]	2012	Family report	1	F	NA	Chinese	M (*COL14A1*)	9	1
Gao [27]	2005	Family report	1	M	47	Chinese	C	14	2
Lienemann [28]	2004	Family report	1	M	49	NA	C	2	1
Asadi [29]	2003	Family report	1	M	75	NA	C	2	1
Emmert [30]	2003	Family report	1	M	76	NA	C	3	1
Martinez-Mir [31]	2003	Family reports (3)	1^b^	M	83	Syrian(1), Arab–Israeli(1), Mexican(1)^d^	C	57	2^e^
Stevens [9]	1996	Family report	1	F	46	Irish	C	49	10
Bennion [8]	1984	Family report	1	M	43	‘white man’	C	8	3
Smith [32]^f^	1970	Cohort study	7	NA^f^	NA^f^	NA^f^	C	NA^f^	NA^f^
Shaffer [33]	1945	Family report	1	M	67	‘colored man’	C	5	1
Neuber [34]	1930	Single case	1	M	67	NA	C	1	1
								** ~ 602**	** ~ 50**
Harjama [35]	2021	Cohort study	9 g	M(4), F(5)	NA	Finnish	M (*AAGAB*)	9	0
Klein [36]	2021	Family report	1	F	68	NA	M (*AAGAB*)	4	0
Pimenta [37]^a^	2019	Family reports (3)	3	F(3)	59–65	NA	M (*AAGAB*)	≥ 6	0
Zamiri [38]	2019	Family reports (16)	16	NA	NA	NA	M (*AAGAB*)	> 30	0
Bukhari [39]	2019	Single case	1	M	59	NA	C	1	0
Monteiro [40]	2019	Family report	1	F	53	Caucasian	C	2	0
Panetta [41]	2017	Family report	1	M	55	Caucasian	C	2	0
Asemota [11]	2016	Single case	1	F	53	NA	C	1	0
Nomura [42]	2015	Family report	1	F	80	Japanese	M (*AAGAB*)	2	0
Li [43]	2014	Family report	1	F	48	Chinese	M (*AAGAB*)	7	0
Pai [44]	2012	Family report	1	M	52	NA	C	2	0
Rapprich [45]	2011	Sngle case	1	M	44	NA	C	1	0
Miljkovic [46]	2009	Family reports (11) Single cases	62 4	M(38), F (28)	NA	Slovenia	C	66	0
Bchetnia [47]	2009	Family reports (5)	5	M(4), F(1)	NA	Tunisian	C	16	0
Cooke [48]	2007	Single case	1	M	50	NA	C	1	0
Erkek [49]	2007	Family report	1	M	41	NA	C	3	0
Oztas [10]	2007	Family report	1	M	70	NA	C	3	0
Kumari [14]	2006	Family report	1	M	8	NA	C	5	0
Kong [50]	2004	Family report	1	F	61	German	C	7	0
Schreiber [51]	1997	Single case	1	NA	56	NA	C	7	0
Hesse [52]	1993	Family report	1	M	48	Caucasian	C	4	0
Rustad [53]	1990	Cohort study^h^	44	M(30), F(14)	NA	black(20), white(18), other(6)	C	44	0
								** ~ 215**	0
In total	1930–2021	Family report (24) Family reports (10) Single cases (6) Cohort study (3) mixture^i^ (2)	1–62 (sum = 280)	M(126) F(98) NA(46)	1–95		C (31) M (12) Mixture^i^ (2)	** ~ 817**	50

Bold marks the sum of the numbers above

*NA* not available, *M* male, *F* female

^a^Conference abstract

^b^Some family/families where no index case is marked

^c^The following information appears in the text ‘*Person marked by asterisk are died by different types of cancers.*’ However, we could not find any asterisk symbols. d) Family 3 (Mexican) was previously reported in an abstract by Davalos et al. [54]

^e^*‘some of his siblings died from unknown types of cancer, according to their* relatives’. However, it is not stated whether his siblings had PPPK1

^f^Report on a group of 47 subjects with palmar lesions, of whom 7 had keratosis only outside the creases [which may be suggestive of PPPK1]. However, descriptive data were only available for the entire group of subjects with palmar lesions and are therefore not entered in the table. The authors report on 3 subjects with cancer (basal cell epithelioma (NA,NA), adenocarcinoma of the prostate (M,NA), lymphoma (NA,NA)), but it is not clear whether these are from the 7 subjects with keratosis only outside the creases or from the entire group.

^g^Only subjects from the cohort with disease-causing variants in *AAGAB* are entered in the table as information on malignancy status was only available for this subgroup of subjects in the study

^h^Rustad et al.[53]: ‘*Most patients denied knowledge of other family members…[with punctate keratosis]…and we did not attempt to examine family members*’.

^i^Single cases and family reports in the same studies.

^j^Single cases and/or family reports with both clinical and molecular genetic verified diagnosis

### Study results related to our research question

We identified 23 studies reporting on individuals with PPPK1 with a history of malignancy and 22 studies reporting on individuals with PPPK1 without a history of malignancies (Table 1 and Additional file 1: Table S2).

The number of index cases with PPPK1 across all 45 included studies was 280 (range 1–62). Adding the number of identified family members with PPPK1 led to a total of 817 individuals with PPPK1. Among these, we identified 50 individuals with PPPK1 and with a history of malignancy. The types of reported malignancies are summarized in Table 2 and in detail in Table S3. In the reported cases with malignancies, both males and females were represented, and the age of the study subjects with PPPK1 ranged from 26 to 94 years, but data on sex and/or age were often not reported (Table 2 and Additional file 1: Table S3).

**Table 2:** Summary of malignancies reported in the 45 included studies

Type of malignancy	Number of individuals	Ages in years
*The type of malignancies (n = 53) reported among 50 individuals with PPPK1*		
Colorectal	7	35, 43,46, 55, 82, NA, NA
Breast	5	46,94,NA,NA,NA
Pancreas	5	65,67,74,80,NA
Prostate	3	71, NA,NA
Hodgkin's lymphoma	3	30,45, 66
Renal	3	26,75, NA
Melanoma	3	50, NA
Squamous cell carcinoma	3	60,76,NA
Basal cell carcinoma	2	NA, NA
Lung	2	NA,NA
Breast and bone	1	NA
Uterus	1	63
Hepatocellular	1	NA
Oesophageal	1	NA
Atypical fibroxanthoma	1	73
Ethmoidal carcinoma	1	65
Myeloma	1	NA
‘Magencarcinom’ [stomach cancer]	1	67
Unknown type	9	NA

Quotations from the included studies illustrating how information on history of malignancies was reported are provided in Additional file 1: Table S4.

### Quality assessment

**Fig. 2:**
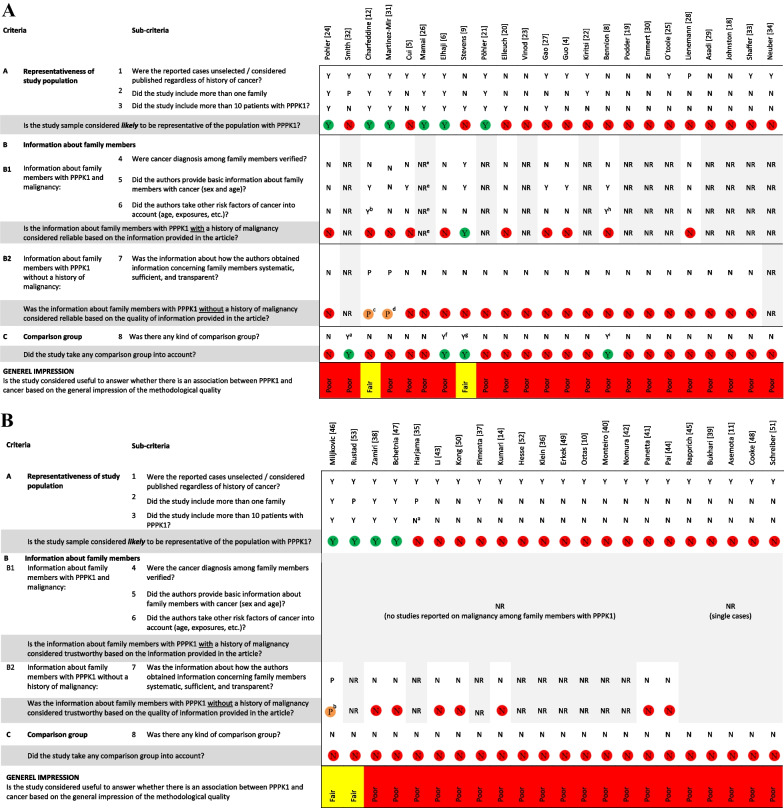
**A** Quality assessment of 23 studies reporting on cases with PPPK1 with a history of malignancy (Y: yes. N: no. P: perhaps. NR: not relevant. (a) Group of patients without palmar lesions. (b) Sex. (c) ‘*all available members were interviewed using a questionnaire including a pedigree drawing in order to link closely related families. The questionnaire was conducted to collect family data…and family history regarding the disease comorbidity*’ [12]. (d) [*affected and a number of unaffected individuals]’…were interviewed and examined by two physicians*’ [31] (e) In the article by Mamai et al., the following information appears in the text ‘Person marked by asterisk are died by different types of cancers’ [26]. However, we could not find any asterisk symbols. (f) Familial cancer in the general Canadian population. (g) Family members without PPPK1. (h) Arsenic exposure (i) Statistical calculation of probability of two rare conditions in two family members). **B** Quality assessment of the 22 studies reporting on individuals with PPPK1 without history of malignancy (Y: yes. N: no. P: perhaps. NR: not relevant. (a) Only subjects from the cohort with disease-causing variants in AAGAB are entered in the figure as information on malignancy status was only available for this subgroup of subjects in the study. (b) ‘*The data were entered into prepared questionnaires* ’[46]).

The included studies were methodologically heterogeneous and could not provide complete valid information to answer our research question on an association between PPPK1 and malignancy. Our evaluation of the methodological quality of each study in relation to our research question is shown in Fig. 2A, B. In only 10 of the 45 studies was the population considered likely to be representative for a broad cohort of patients with PPPK1 (criterion A). Information on malignancies among family members was considered reliable in only one study based on basic information about sex, age, and verification of cancer diagnosis (Criterion B1). In none of the studies was the information about family members with PPPK1 without a history of malignancy considered reliable based on the quality of information provided in the article (Criterion B2), and only four studies took any kind of comparison group into account (Criterion C). Based on the predefined quality criteria, our general impression of the methodological quality of the included articles was poor (n = 41), fair (n = 4), and good (n = 0) in terms of whether each study was considered useful in answering our research question.

## Discussion

In this systematic review, we evaluated the current evidence in the literature on the hypothesis proposed by some [6, 8, 9, 28] that PPPK1 is associated with an increased risk of malignancies. We found the methodological quality of the existing literature of to poor quality to definitely confirm or reject such an association. However, based on our study, we find the suggested association should be considered with caution and find it highly questionable to recommend surveillance for malignancies due to the presence of PPPK1 as a risk factor in itself. In the presence of a positive personal or familial history of cancer in people with PPPK1 it is advised (i) to explore the potential co-existence of PPPK1 with a known cancer-predisposition syndrome, and, if no such syndrome is identified (ii) to recommend the affected individuals to simply follow standard guidelines for monitoring the cancer risk in the general population according to age and sex. By this approach, individuals with PPPK1 can receive appropriate attention and monitoring, without assuming an inherent increased risk solely based on the presence of PPPK1.

We identified 45 studies reporting on patients with PPPK1 with *or* without a history of malignancies, covering more than 800 individuals with PPPK1. However, most of the studies had other objectives that did not concur with our research question and/or did not have an appropriate design for data. We found that most data about history of malignancy had insufficient quality to be synthesized without considerable reservations. However, we identified only 50 patients with PPPK1 with malignancies among the quite high number of individuals with this rare disease. Given the frequency of cancer in the general population, these numbers do not indicate a positive association between PPPK1 and malignancy. In addition, the reported malignancies were of various types, and the most frequent cancers (colorectal, prostate, and breast) are also those most frequently seen in the general population. This does not suggest any associations of PPPK1 with specific types of cancer as seen in several other cancer-predisposition syndromes. Furthermore, most cases with PPPK1 and malignancy (41 out of 50) were either above 60 years of age (*n* = 21) or the information on age was not available (*n* = 20).

### Considerations on methodology in the reviewed literature

Only a few studies had patient populations that we considered likely to be representative for the population with PPPK1 based on our predefined criteria. A main concern was the risk of selection bias, if reports were more likely to be published because of a co-presence of PPPK1 and malignancy and thereby a bias towards a positive association. Another concern was studies including only one family as there is a risk of two different diseases co-segregating in the same family. This phenomenon has been shown in a family studied by Blanchet-Bardon et al. [55], in which an increased risk of breast and ovarian cancer among family members with diffuse PPK was suggested. Later genetic analysis of the family showed that the diffuse PPK was due to a disease-causing variant in *KRT9,* whereas the breast and ovarian cancer was due to a disease-causing variant in *BRCA1*. This is an important example of how an association can be explained by variants in genes located in the same area of the genome and thereby co-segregating [56, 57]. This phenomenon may also be considered in the study by Stevens et al. [9], which was one of the first to suggest a possible association between PPPK1 and malignancy, and is one of the few studies we evaluated as being ‘fairly’ useful; however, it only includes one family.

Most of the studies did not indicate whether malignancy in family members was verified, and several studies did not state sex and/or age of family members with PPPK1 and a cancer diagnosis, despite this being essential information in view of age being a major risk factor for malignancy. Furthermore, there was considerable inconsistency across studies regarding the information provided on family members (Additional file 1: Table S4). While some studies explicitly stated the presence or absence of family members with malignancy, others only provided information limited to their index cases. Additionally, only a few studies indicated how information on family members was obtained, making it difficult to determine whether, especially, the absence of reported malignancy was due to a true absence or simply a lack of available information. Another consideration was whether family members without a reported history of malignancy were of a sufficient age to allow for an accurate evaluation of the overall cancer risk. Therefore, improved reporting standards is needed in future studies if information of family members should be included in analysis.

Finally, the majority of the included studies did id not incorporate any comparison group. This may be because they had other objectives or did not report on malignancies, but, nevertheless, many of these studies still commented on a potential association between PPPK1 and cancer. Four studies [6, 8, 9, 32] included some kind of comparison group, but the control group must be carefully selected to answer whether patients with PPPK1 have an increased risk of cancer, and this was not always the case.

### Methodological considerations of our own review

Our search strategy had several strengths. First, we supplemented the search in common literature databases with a search in the Human Gene Mutation Database (HGMD), a well-known genetic database, thus ensuring that all published reports on subjects with disease-causing variants in relevant genes were included. Second, we screened the reference lists of the included studies, which led to a small number of additional studies being identified. Instead of simply incorporating these studies, we evaluated why they were absent in our initial search and identified two additional search terms used as synonyms for ‘punctate’. After adjusting the search, the screening of the reference list led to only two extra studies [20, 23], indicating that the final search strategy was satisfactory.

Another strength was the evaluation of the methodological quality of the included studies in relation to *our* research question. This proved to be important as it led to the conclusion that the methodological quality was too poor to synthesize data. We might have missed this conclusion if we had only summarized the available data from the literature. Finally, the review was strengthened by all steps (screening, data extraction, and quality assessment) being done by two authors independently of each other.

There are also some limitations in this review, mostly related to PPPK1 being a rare disease. The main weakness was that we were unable to find a relevant, validated checklist for the quality assessment as the included studies mostly consisted of family reports. We designed our own checklist with criteria we considered relevant, but other research teams might have designed this differently. This may have led to different quality assessments, but we doubt that it would have led to other conclusions. Another limitation was the language restrictions although the screening of titles and abstracts suggested that use of other languages would most likely not change the overall conclusions.

### Recommendations for future studies

The clear limitations identified in the current literature make it relevant to discuss how future studies should be designed to achieve sufficient methodological quality to answer our research question. To reach a sufficient number of study subjects with this rare disease a multicentre study would be needed. This could be either a retrospective case–control study comparing a number of case subjects with PPPK1 and malignancies to a group of matched control subjects without PPPK1 or a prospective case–control study with a very long observation time. However, the main challenge (even in an international multicentre study) would be to include a sufficient number of cases with PPPK1 due to the rarity of the disease. For this reason, it might be necessary to incorporate information about family members with PPPK1, but it would be crucial to be systematic and transparent in the approach of obtaining information about family members to ensure valid information.

Our review illustrates some of the concerns that may arise when early studies point out a possible risk based on one or few families. It may create bias in the literature thereafter as authors may subsequently tend to focus on and publish about individuals with malignancies, and it becomes difficult to reject an association despite a high number of reported cases. It is, therefore, of utmost importance to conduct well-designed studies that have clear hypotheses and take into account the methodological observations discussed above before drawing conclusions that can be highly detrimental to patients, who will become worried for—possibly—no reason.

## Conclusions

This systematic review revealed that the current literature on a possible association between punctate palmoplantar keratoderma (PPPK1) and increased risk of malignancy is insufficient in terms of methodology to draw definite conclusions. We did not find adequate evidence confirming an association and find it questionable whether individuals with PPPK1 should be offered surveillance for malignancies. Well-designed studies are necessary to provide evidence-based guidance to clinicians and patients and to more firmly accept or reject the hypothesis that PPPK1 is associated with an increased risk of malignancy.

### Supplementary Information

**Additional file 1**. **Table S1:** Custom-made tool for assessing the methodological quality of the included studies. **Table S2:** Additional descriptive data from the included studies. **Table S3**: Reported type of malignancy and sex, age at diagnosis in the listed studies.** Table S4:** Examples on how information about history or no history of malignancies was provided in the included studies.

## Data Availability

All data supporting the findings of this study are available within the paper and it’s Supplementary Information files.

## Abbreviations

PPK: Palmoplantar keratoderma
PPPK1: Punctate palmoplantar keratoderma type 1
PRISMA: Preferred reporting items for systematic reviews and meta-analyses
HGMD: Human gene mutation database
