# Supplementary material for: Is punctate palmoplantar keratoderma type 1 associated with malignancy? A systematic review of the literature
Source: Orphanet J Rare Dis. 2023 Sep 13;18:290. doi: 10.1186/s13023-023-02862-8 (PMC10500882; doi:10.1186/s13023-023-02862-8)
Supplement: Supplementary file 1 — Additional file 1. Table S1: Custom-made tool for assessing the methodological quality of the included studies. Table S2: Additional descriptive data from the included studies. Table S3: Reported type of malignancy and sex, age at diagnosis in the listed studies. Table S4: Examples on how information about history or no history of malignancies was provided in the included studies. [file 13023_2023_2862_MOESM1_ESM.docx]

**SUPPLEMENTARY**

| **Table S1** Custom-made tool for assessing the methodological quality of the included studies | | | |
| --- | --- | --- | --- |
| **Criteria and sub-criteria** | | | |
| Criterion A. Representativeness of study population: | | | |
| 1. Sub-criterion: Were the reported cases unselected / considered published regardless of history of malignancies?  2. Sub-criterion: Did the study include more than one family?  3. Sub-criterion: Did the study include more than 10 individuals with PPPK1? | |  |  |
| Assessment of criterion A: Is the study sample considered likely to be representative of the population with PPPK1? | |  |  |
| Criterion B. Information about family members | | | |
|  | | | Criterion B1. Information about family members with PPPK1 and malignancy |
| 4. Sub-criterion: Was malignancy diagnosis among family members verified?  5. Sub-criterion: Did the authors provide basic information about family members with malignancies (sex and age)?  6. Sub-criterion. Did the authors take other risk factors for malignancy into account (age, exposures, etc.) | |  |  |
|  | | | Assessment of criterion B1: Is the information about family members with PPPK1 with a history of malignancy considered reliable based on the information provided in the article? |
|  | | | Criterion B2. Information about family members without history of malignancies |
| 7. Sub-criterion: Was the information about how the authors obtained information about family members without reported history of malignancy systematic, sufficient, and transparent? | | | |
|  |  | | Assessment of criterion B2: Was the information about family members with PPPK1 without reported history of malignancy considered reliable based on the quality of information provided in the study? |
| Criterion C. Comparison group | | | |
| 8. Sub-criterion: Was there any kind of comparison group? | |  |  |
| Assessment of criterion C: Did the study take any comparison group into account? | |  |  |
| **GENERAL IMPRESSION**  Is the study considered useful to answer whether there is an association between PPPK1 and malignancy based on the general impression of the methodological quality? | | | |

**TABLE S2**

|  |  |  | |
| --- | --- | --- | --- |
| **Table S2:** Additional descriptive data from the included studies. | | | |
| **Author** | **Consanguinity** | **Disease duration , index patient** | |
|  | no. of families | Years with PPPK1 | Age at onset (no. of cases) |
| Harjama [35] | NA | - | 0-10 (2), 11-20 (4), 21-30 (2), >31 (1) |
| Klein [36] | NA | 40 |  |
| Elhaji [6] | NA | - | 20-55^a^ |
| Johnston [18] | NA | NA | |
| Pimenta [37] | Y(1),N(2) | 10 to 14 | - |
| Zamiri [38] | NA | - | 8-55 |
| Bukhari [39] | N | 25 | - |
| Monteiro [40] | NA | 10 | - |
| Panetta [41] | NA | 9 | - |
| Charfeddine [12] | Y (6),NA (2) |  | 10 - 5^th^ decade of life ^b^ |
| Asemota [11] | NA | 13 | - |
| Podder [19] | NA | - | 17 |
| Nomura [42] | NA | - | 1^st^ decade of life |
| Elleuch [20] | Y |  | 30 |
| Li [43] | NA | - | 18 |
| Cui [5] | NA | NA | |
| Pöhler [21] | NA | 15-30 | - |
| Kiritsi [22] | NA | - | Childhood-30 |
| Vinod [23] | NA | 45^c^ | 18 ^c^ |
| Pohler [24] | NA(17),Y(1) | - | 1^st^ to 2th decade of life |
| Pai [44] | N | 30 | - |
| O'Toole [25] | NA | - | 15 |
| Mamai [26] | Y(1),NA(2) | NA^d^ | |
| Guo [4] | NA | - | 10-35 |
| Rapprich [45] | NA | 15 | - |
| Miljkovic [46] | NA | first year of life (6), before age ten (3), 20-40 years (10), NA (47) | |
| Bchetnia [12] | Y(3),NA(2) | - | 10 – fifth decade |
| Cooke [47] | NA | ‘several years’ - | |
| Erkek [48] | NA | - | ‘after puberty’ |
| Oztas [10] | NA | >40 | - |
| Kumari [14] | NA | - | 6 month |
| Gao [27] | NA |  | 25 |
| Lienemann [28] | NA | - | ‘since he was teenager’ |
| Kong [49] | NA | - | 15 |
| Asadi [29] | NA | - | ‘many years’ |
| Emmert [30] | N | - | third decade |
| Martinez-Mir [31] | Y (2),NA(1) | NA ^i^ | |
| Schreiber [50] | NA | 8 | - |
| Stevens [9] | NA | - | ‘in her twenties’ |
| Hesse [51] | NA |  | adolescence |
| Rustad [52] | NA | - | 15-68 |
| Bennion [8] | NA | 27 | - |
| Smith [32] | NA | NA | |
| Shaffer [33] | NA | ‘as long as he could remember’ | |
| Neuber [34] | NA | 20-22 ^c^ | 45-47 ^c^ |
| a) Except one patient from early childhood. b) There was a discrepancy between information in the pedigree and the text in the article. Information from the text is entered in the table. c) Both numbers are reported in the study. d) Duration stated for all subjects but not explicit for the index cases. | | | |

**TABLE S3**

| **Table S3:** Reported type of malignancy and sex,age at diagnosis in the listed studies. | |
| --- | --- |
| Pohler [24] | ‘*a few cases of cancer were reported in the larger families’* |
| Charfeddine [12] | prostatic carcinoma (M,71) |
| Martinez-Mir [31] | breast cancer (NA,94), pancreatic carcinoma (M,NA). |
| Cui [4] | unknown (M (3) / F (2) ,NA) |
|  | unknown (NA,NA) x 3 |
| Mamai [26] | ? ^a^ |
| Elhaji [6] ^b^ | colon (F,NA), multiple myeloma (F,NA), breast (F,NA)(n=2), prostate (M,NA), esophageal (M,NA), basal cell (M,NA), renal cell (M,NA), breast and bone (F,NA) |
| Stevens [9] | colon (F,82) and (F,46), pancreas (F,80) and (F,74), renal cell carcinoma (M,75), leiomyosarcoma of the uterus (F,63), Hodgkin lymphoma (F,30) and (M,45), breast (F,46), renal adenocarcinoma (M,26) |
| Pöhler [21] | cutaneous squamous cell carcinoma on the hand (M,NA) + basal cell carcinoma on the nose (M,NA) ^c^ |
| Elleuch [20] | hepatocellular carcinoma (F,NA), melanoma (M, NA), colorectal cancer (M,NA) |
| Gao [27] | lung carcinoma (M,50) and unknown (M,NA) |
| Guo [4] | colonic adenocarcinoma (F, 35) |
| Kiritsi [22] | breast cancer (F,NA) |
| Bennion [8] | adenocarcinoma of the colon (M,43), adenocarcinoma of the colon (F,55), pancreatic carcinoma (M,65) |
| Vinod [23] | squamous cell carcinoma of anterior chest wall (60,M) + ethmoidal carcinoma (65,M) ^c^ |
| Podder [19] | Hodgkin lymphoma (M,66) |
| Shaffer [33] | carcinoma of the pancreas (M,67) |
| Emmert [30] | squamous cell carcinoma on the scalp (M,76) |
| O'Toole [25] | melanoma (F,50) |
| Lienemann [28] | lung cancer (M,NA) |
| Asadi [29] | prostate cancer (M,NA), atypical fibroxanthoma (M,72) ^c^ |
| Johnston [18] | malignant melanoma (F,80) |
| Neuber [34] | ‘magencarcinom’ [stomach cancer], (M,67) |
| Smith [32] | NA^d^ |
| a) In the article by Mamai et al. [26], the following information appears in the text ‘*Person marked by asterisk are died by different types of cancers.*’ However, we could not find any asterisk symbols. b) In the article by Elhaji et al. [6] there seems to be a difference between malignancies reported in their table and in the pedigrees. We have inserted the information provided in the pedigrees. c) Same patient had both cancers. d) Smith et al. [32]: The authors report on 3 subjects with cancer (basal cell epithelioma (NA,NA), adenocarcinoma of the prostate (M,NA), lymphoma (NA,NA)), but it is not clear whether these are from the 7 subjects with punctate keratoses [may be suspected for PPPK1] or from the entire patient group. | |

**TABLE** S4

| **Author** | **Examples on how information about history of malignancies was provided** |
| --- | --- |
| Pohler [24] | *‘Although a few cases of cancer were reported in the larger families studied here, we did not observe cosegregation with the AAGAB mutations’* |
| Charfeddine [12] | *‘Moreover, patient II-7 in family PPK8, aged 77 years, was diagnosed with prostatic carcinoma of about 6-year duration. There was a family history of PPPK-BFB over three generations; none of these members were diagnosed with any malignancy (according to the information supplied by examined family members).* |
| Martinez-Mir [31] | *‘The only case was that of an affected patient who died from breast cancer at the age of 94 years.’ [family 1 and 2]*  *‘The proband died from pancreatic carcinoma and some of his siblings died from unknown types of cancer, according to their relatives.´ [family 3]* |
| Cui [4] | *‘In addition, we identified thec.481C>T mutation in exon 5 in one patient with cancer from Family 24, whereas none of three sporadic patients with cancer carried mutations. There appeared to be no genetic link between AAGAB mutations and cancer.’* |
| Mamai [26] | *‘Fig. 1: …person marked by asterisk are died by different types of cancers’* |
| Elhaji [6] | *‘Different cancers were reported in 10 families (Table 1 and Supplemental Figure S1). These include melanoma, esophageal, breast, pancreatic, bone, lung, throat, prostate, bowel, and brain as well as renal and basal cell carcinomas. We were unable to obtain cancer history for all members of all families and did not confirm the pathological diagnosis of cancer in the affected families.’* |
| Stevens [9] | *‘A striking association between these PPK lesions and cancer was noted within the family, with a number of tumours developing before the age of 50. The family pedigree (part shown in Figure 1) comprises over 320 individuals, and both affected and unaffected members who developed malignancy are shown in the family tree.’*  *‘The pathological diagnosis of all known malignancies was checked with the diagnostic pathologist.’* |
| Pöhler [21] | *‘… malignant neoplasms were not prevalent in the six kindreds reported here’* |
| Elleuch [20] | *‘The research of similar cases in the family had revealed the presence of the KPP 1 in many other family members over four generations. It was associated with colorectal cancer, hepatocellular carcinoma and melanoma in different family members (Figure 5).’* |
| Gao [27] | *‘In this family, individual III:3 had died from lung carcinoma at the age of 50 years, and his father (individual II:1) had also died from an unknown type of cancer, according to other family members. However, we did not observe an increased prevalence of malignancy in other family members’*  *‘Two affected members of our study family had had carcinomas.’* |
| Guo [4] | *‘One affected member (II10) in PPPK703 developed colonic adenocarcinoma when she was 35-years-old.’* |
| Kiritsi [22] | *‘Interestingly, patient 4 reported that the lesions had vanished during chemotherapy for breast cancer, and reappeared gradually after completion of the therapy (Figure 1d).’* |
| Bennion [8] | *‘Other family members had evidence of punctate keratoderma, but no other cancers have been discovered to date’* |
| Vinod [23] | *‘There was a strong family history with 7 members affected over 4 generations; none of these members were diagnosed to have any malignancy.’* |
| Podder [19] | *‘The histopathological findings were consistent with Type I hereditary punctate palmo-plantar keratoderma (PPKD) (Buschke-Fischer-Brauer keratoderma) associated with Hodgkin's lymphoma.’* |
| Shaffer [33] | *‘…male who entered the hospital with an inoperable carcinoma of the pancreas…’* |
| Emmert [30] | *‘In the family reported herein, only the 76-year-old patient developed a squamous cell carcinoma on his scalp.’* |
| O'Toole [25] | *‘Aside from having a melanoma excised from her right thigh in 2008, there was otherwise no personal history of cancer’*  *‘The family history was significant for mesothelioma in the patient's father secondary to asbestos exposure at a steel factory. There was no other family history of cancer.’* |
| Lienemann [28] | *‘His father also had this skin disorder, which coincidentally cleared after 2 courses of chemotherapy consisting of 5-FU and cisplatin to treat this lung cancer’* |
| Asadi [29] | *‘A 75-year-old man with a history of prostatic carcinoma and atypical fibroxanthoma reports a long-standing history of 1-2 mm depressed, hyperkeratotic papules on the palms. His mother suffered a similar condition.’* |
| Johnston [18] | *‘…confirmed an ulcerated acral lentiginous malignant melanoma’* |
| Neuber [34] | *‘Nur vor ½ Jahr traten Verdauungsstörungen und Magenschmerzen auf (Carcinom).’*  *[Only for ½ year, indigestion and abdominal pain (carcinoma) appeared]* |
| Smith [32] | *‘Our patients did not have an elaborate cancer survey, but their charts were reviewed for a diagnosis of malignancy. ’* |
|  |  |
| **Author** | **Examples on how information about no history of malignancies was provided** |
| Miljkovic [46] | *‘No malignancies were observed, nor were such data revealed in patients’ histories’* |
| Rustad [52] | *‘none had history of internal malignancy’* |
| Zamiri [38] * | *‘there was no association with malignancy or diabetes mellitus in affected members of any of the families’* |
| Bchetnia [12] | *‘we did not observe any type of malignancy associated with type I punctate PPK on the examined Tunisian patients.’* |
| Harjama [35] | *‘None of our AAGAB patients had concomitant malignancies’* |
| Li [43] | *‘The patients in this family could not be associated with malignancy’* |
| Kong [49] | *‘no malignancies have been detected to date...all family members in the pedigree were generally healthy without any reported history of malignancies’* |
| Pimenta [37] | *‘They denied exposure to arsenic or personal history of neoplasia’* |
| Kumari [14] | *‘There was no history of any malignancy in any of the affected family members.’* |
| Hesse [51] | *‘gastrointestinal disorders (colonic adenocarcinoma, gastro-duodenal ulcers) have been reported in association with keratosis punctata, but were not present in our case.’* |
| Klein [36] | *‘In this patient, the results of recent chest radiography, upper endoscopy, colonoscopy, Papanicolaou testing, and mammography were unremarkable.’* |
| Erkek [48] | *‘In our patient, there was no evidence of malignancy or pigmentary alterations.’* |
| Oztas [10] | *‘An extensive search for associated malignancy was undertaken in our patient. However, no evidence of malignancy was detected’* |
| Monteiro [40] | *‘A thorough work-up was carried out leading to exclusion of malignant neoplasms’* |
| Nomura [42] | *‘Although our patient has not developed any malignancies’* |
| Panetta [41] | *‘The familial and personal medical history was negative for malignancies … in both patients’* |
| Pai [44] | *‘No personal or family history of diabetes, hypertension or malignancy was present’* |
| Rapprich [45] | *‘Klinische Hinweise für eine Tumorerkrankung ergaben sich nach Labordiagnostik, Oberbauchsonographie und Röntgen-Thorax nicht’*  *[Clinical indications of a tumor disease were not found after laboratory diagnostics, upper abdominal sonography and chest X-ray]* |
| Bukhari [39] | *‘However, our patient has none of these malignancies’* |
| Asemota [11] | *‘The patient was up to date on her age-appropriate malignancy screening’* |
| Cooke [47] | *‘Further investigations to exclude malignancy did not reveal any significant abnormalities’* |
| Schreiber [50] | *‘Auch bei intensive mehrmaliger Durchuntersuchung fand sich während der nunmehr einjährigen Beobachtungszeit kein Hinweis auf ein bösartiges Tumorwachstum’*  *[‘Even with intensive repeated examinations, there was no evidence of malignant tumor growth during the now one-year observation period’]* |
